# Inter-Regional Hospital Patients’ Mobility in Italy

**DOI:** 10.3390/healthcare9091182

**Published:** 2021-09-08

**Authors:** Nicola Nante, Giovanni Guarducci, Carlotta Lorenzini, Gabriele Messina, Flavia Carle, Simona Carbone, Andrea Urbani

**Affiliations:** 1 Department of Molecular and Developmental Medicine, University of Siena, 53100 Siena, Italy; gabriele.messina@unisi.it; 2 Post Graduate School of Public Health, University of Siena, 53100 Siena, Italy; giovanni.guarducc@student.unisi.it (G.G.); lorenzini27@student.unisi.it (C.L.); 3 Centre for Healthcare Research and Pharmacoepidemiology, Polytechnic University of Marche Region, 60121 Ancona, Italy; f.carle@univpm.it; 4 General Directorate for Health Planning, Ministry of Health, 01144 Rome, Italy; s.carbone@sanita.it (S.C.); dp.segreteria@sanita.it (A.U.)

**Keywords:** patients’ mobility, Italian National Health Service (NHS), Gandy’s Nomogram, regional hospital networks

## Abstract

Background: The federalization of the Italian National Health Service (NHS) gave administrative, financial, and managerial independence to regions. They are in reciprocal competition according to the “quasi-market” model. A network of independent providers replaced the state monopoly. The NHS, based on the Beveridge model in which citizens are free to choose their place of treatment, was consolidated. The aim of our research was to analyze the fulfillment of need for hospital services on site and patients’ migration to hospitals of other regions. Material and Methods: We analyzed data from 2013 to 2017 of Hospital Discharge Cards (HDCs) provided by the Ministry of Health. The subjects of the analysis (catchment areas) were the hospital networks of every Italian region. The study of flows was developed through Internal Demand Satisfaction, Attraction, Escape, Attraction, Absorption, and Escape Production indexes. Graphic representations were produced using Gandy’s Nomogram and Qgis software. Results: In the studied period, the mean number of mobility admission was 678.659 ± 3.388, with an increase of 0.90%; in particular, the trend for ordinary regime increased 1.17%. Regions of central/northern Italy have attracted more than 60% of the escapes of the southern ones. Gandy’s Nomogram showed that only nine regions had optimal public hospital planning (Lombardy, Autonomous Province of Bolzano, Veneto, Friuli V.G., Emilia-Romagna, Tuscany, Umbria, Latium and Molise). Conclusion: The central/northern regions appear more able to meet the care needs of their citizens and to attract patients than the southern ones.

## 1. Introduction

Between the second and third millennium, socioeconomic, demographic, cultural, and epidemiologic transformations occurred, which significantly affected people’s health and healthcare. As result, life expectancy increased but, also consequently, the prevalence of chronic diseases. The individualistic culture created expectations regarding well-being; people started to spend more on drugs and services. At the same time, the development and the affordability of technologies affect the problems of self-care and self-diagnosis. The constant improvement in the accessibility of transportation and information has increased the phenomenon of willingness to move national and international distances in search of better treatments [1,2]. Medical care offered to patients willing to travel for care includes a wide variety of services such as bio-ethical treatments (fertility, transplantation, and stem cell therapies), diagnostic tests, dental care, and surgery (cosmetic, orthopedic, bariatric, ophthalmology, and heart). In the recent years, healthcare mobility has developed rapidly and grown in social importance: especially in Europe, where a relatively large number of people are already receiving cross-border healthcare for elective or acute treatment, due to the European Union actively supporting patients in seeking cross-border healthcare by disseminating information through brochures, fact sheets and specialized contact points [3,4,5,6].

Healthcare mobility is a complex migratory phenomenon of patients who benefit from the healthcare services far from their place of residence. With EU legislation, the analysis of these flows at a national—but also international—level involves aspects concerning quality of services (real/perceived) and equity of access to care; it also has important economic implications [7,8,9,10]. The uniform accessibility to services and their quality are a primary aim of the Italian National Health Service (NHS). Healthcare mobility, according to Italian Law, can be considered an expression of inviolable rights of health and freedom to choose a physician and place of care, within objective limits of health services organization and availability of resources. Healthcare reforms in 1992 and 1993 further extended the freedom of choice. With the corporatization of Local Health Units (LHSs) and consequent empowerment of health planning in terms of economic–financial budget, as well as the end of the process of health federalism, the phenomenon of health migration is playing an increasingly important role. It appears inevitably connected to the different "speed" of regional health systems, which presents both quantitative and qualitative differences in the supply of health services. In this context, the development of new information media and transport networks as well as the increase in healthcare provisions and technologies is crucial [11,12,13,14]. All these tools simultaneously contribute to offer to the patient the possibility of deciding more consciously where to be treated, since the ELC (Essential Levels of Care) can be provided throughout all national territory by public, private equivalent, and accredited private structures, whereby regions stipulate agreements or contracts with them. From this point of view, healthcare mobility can be considered an opportunity, since it meets, as much as possible, the needs of assistance to guarantee freedom of choice regarding place of care. Therefore, we can deduce that health planning, in order to achieve an equitable distribution of services offered in the territory, should take its cue from patients’ choices about which place they considered most appropriate to their needs. It is clear that both the qualitative and quantitative uniformity of health networks represents the squaring of the circle [15,16,17,18].

Patients’ mobility from our own region is a negative, indirect, and implicit index of the quality and quantity of services provided by their region [14,19]. In fact, moving patients “judge” the nosocomial and health services: hence, the expression coined in 1956 by the economist Charles Mills Tiebout: “Patients vote with their feet” [20]. Several factors play a role in the patients’ choice, including the heterogeneity of health services and the complexity of social, demographic, and economic factors. It has also been observed that the choice of structure is influenced by several factors such as a high individual and family income, propensity to travel, level of education, patient’s age, type of pathology, and frequency of recourse to hospital care [21,22,23]. In fact, residents of less populated areas, especially if they are frail elderly, where the competition between healthcare facilities is weak, tend to choose what the system offers. However, hospitals (especially private ones), usually located in populated areas, generally offer longer lengths of stay and more flexible waiting lists to attract this type of patient [24,25,26]. It has been proved [27] that a higher income is associated with a greater willingness to travel, although there is a saturation point (distance) beyond which the income effect is greatly reduced [28]. However, variables such as lower level of education, older age, and female gender negatively affect the choice of the healthcare facility and the propensity to travel long distances for care [29,30,31]. 

In addition, “severe illnesses”, which might imply less frequent but regular hospital visits (such as for chemotherapy or renal dialysis), cause lower willingness to travel [32]. Other studies [33,34] show that patients are willing to travel to distant hospitals with strong reputations for cancer conditions, especially those who are younger and with higher incomes. While for other conditions such as non-urgent hip replacement [35] and cataracts [36], patients prefer hospitals located nearby with better performance and shorter wait times. Another research study has quantified the importance of several factors in choosing a hospital for surgery. It proved that the reputation of the department and direct knowledge of physicians working there have a significantly greater weight than distance or waiting time in determining “healthcare migration” [19]. 

Therefore, the analysis of healthcare mobility (in particular hospital) is a fundamental tool for policy planning, to monitor the activities carried out by different healthcare providers. This phenomenon is linked to aspects such as equity, trust in patients, and the reputation of the structures. It synthesizes psychometric and econometric data, which can be divided into information from the MACRO level (state or regional health policy), MESO level (management of companies/hospital facilities), and MICRO level (management of wards/management control) [11,23,37,38].

The aim of our research is to analyze, through healthcare mobility, how Italian National Health Service pursues equity of access to services and their quality. The findings of the current study can be useful to identify points of strength and weakness of hospital care and areas of potential improvement intervention.

## 2. Materials and Methods

### 2.1. Data and Catchment Areas

We collected data from 2013 to 2017 from the Database of Hospital Discharge Cards (HDCs) of the Ministry of Health, upon specific request, because data published on the Ministry’s online website does not provide information about the type of hospital or Major Diagnostic Categories (MDCs).

We included acute hospitalizations of Italian patients, and we excluded discharges of patients residing in other states admitted in Italian hospitals and hospitalizations of Italian citizens in foreign hospitals. The study of healthcare mobility flows relative to single Italian regions was carried out from data relative to the hospitalization of R = Residents, A = Attractions, and E = Escapes. 

### 2.2. Analysis Techniques

For all regions, we calculated the following mobility indicators:

(a). The Internal Demand Satisfaction Index (IDSI), which indicates the ratio between the difference between Attractions (A) and Escapes (E) and the sum of Residents (R) and Attractions (A), in formula:
  IDSI = A − E/(R + A) × 100.(1)

A Region with a negative IDSI supplies every year an unsatisfactory number of admissions to meet, completely, the overall demand of its own inhabitants. Positive values of ISDI show that the region is able to satisfy the overall demand expressed by its own inhabitants entirely. The ISDI gives important information related to the “size” of the healthcare supply compared with the effective demand expressed by its own population [39].

(b). The Attraction Index (AI), which indicates the percentage of Attraction (A) out of the total number of hospitalizations in the Region (A + R), in formula:
  AI = A/(R + A) × 100.(2)
(c). The Escape Index (EI), which indicates the percentage of Escapes (E) out of the total number of discharges of residents (R + E), in formula:
  EI = E/(R + E) × 100.(3)

The Attraction Index measures the capacity of a region to attract patients from other regions, while the Escape Index quantifies the propensity of patients to leave from their own region of residence [40,41].

(d). The Attractions Absorption Index (AAI) indicates the percentage of discharges of Attracted from region Xi (with i = 1, ..., 21), out of total Attractions of all regions, in the following formula:
  AAI Xi = A Xi/Tot. A × 100(4)
  where AAI Xi = Attractions Absorption Index of region Xi; A Xi = Attraction from region Xi; and Tot. A = Total Attraction of all regions.
(e). The Escape Production Index (EPI) indicates the percentage of Escapes from region Xi (with i = 1,...,21), out of the total Escapes of all regions, in the following formula:
  IPE Xi = E Xi/Tot. E × 100(5)
  where IPE Xi = Escape Production Index of region Xi; E Xi = Escapes from region Xi; Tot. E = total Escapes of all regions.

The Attraction Absorption and Escape Production Indexes give an immediate representation of the mobility volume absorbed and generated by each region [41].

Through Gandy’s Nomogram, we have processed data for each single region to represent, briefly and graphically, the access to hospital by residents and non-residents [42,43]. It is a squared area with the side of 100 placed in a Cartesian plan:

- The X value indicates Residents (R) out of Residents (R) plus the Attractions (A):
  X = R/(R + A) × 100.(6)
- The Y value indicates Residents (R) out of Residents (R) plus the Escapes (E):
  Y = R/(R + E) × 100.(7)

From 100 to 0, along the X-axis, the power of Attraction increases, while along the Y-axis, Escapes to other regions increase. The Cartesian plan may be further divided into four squares by two lines, parallel to the axis, which takes the origin at X = 0; Y = 50 and X = 50; Y = 0.

The diagonal that originates from the O point (X = 0; Y = 0) and ends at the W point (X = 100; Y = 100) splits the plan in an upper part where the Y value is larger than the X one, in which there are more Attractions (A) than Escapes (E), and a lower one with an opposite situation. The points on the diagonal have the same value either for Escapes or for Attractions, which are null in the W point and maximum in the O point.

The four above-mentioned quadrants show a different balance between Escapes and Attractions:

- Regions placed in the upper left quadrant have a number of residents’ admissions higher than Escapes and, at the same time, lower than Attractions. This condition characterizes regions as “market oriented” (E < R < A), which are able to get more funds because their hospitals admit more patients from other regions than patients from their own. The point (X = 0, Y = 100) identifies the paradoxical condition in which hospitals of a region admit only patients from other regions and there are no Escapes.
- The upper right quadrant is parted in two areas: a and b. In the first one, the residents’ admissions are higher than Attractions and the latter are higher than Escapes (E < A < R). In the second area, residents’ admissions are higher than Escapes, but the latter are higher than attracted (A < E < R).

In these two areas, there are hospitals that satisfy (in a more or less appropriate way depending on their position) the healthcare needs in their region.

- In the lower left quadrant, a diagonal divided it in two areas. Both of them have a lower number of residents’ admissions, exceeded by Escapes and Attractions: in the upper area, Escapes are lower than Attractions (R < E < A) and in the lower, we have an opposite situation (R < A < E).
- The lower right quadrant shows regions where residents’ admissions are lower than Escapes and higher than Attractions (A < R < E).

Finally, in order to represent the various regional indexes, described above, we made, using Quantum Gis software version 2.16.3 (Open Source Geospatial Foundation Project) [44] cartographic maps with six scales of different color intensity. The stratification into six levels was based on percentiles (≤10%; between 11% and 25%; between 26% and 50%; between 51% and 75%; between 76% and 90%; and finally ≥91%). We assessed the normality of distributions analyzed by Shapiro–Wilk’s test, Mann–Whitney’s test was used to compare day hospital and ordinary admissions, Spearman’s test was used to assess the correlation between the rate of hospitalization and number of beds, and then for the correlation between mobility indicators. Cuzick’s test was used to assess trends over time of X and Y values of Gandy’s Nomogram. Statistical analyses were carried out with STATA software SE/14.0 (StataCorp LLC, Texas USA). Differences were considered at a statistically significant level of 95% (*p* < 0.05).

## 3. Results

Table 1 shows admissions in ordinary and day hospital regime from 2013 to 2017, divided in residents and mobility (escapes/attractions).

In Italy, from 2013 to 2017, the average number of annual admissions to hospital, for acute cases, ordinary (ORD) and day hospital (DH), were 8,314,340 ± 363,415, with an important decrease from 2013 (maximum value: 8,845,127) to 2016 (minimum value: 7,895,947) and a slight increase in 2017 sustained by day hospital. The number of mobility hospitalizations averaged 678,659 ± 3388, with an upward trend until 2016 and a slight decrease in 2017.

In the same period in mobility, the average number for ordinary hospitalization was 502,223 ± 4935, while for day hospital, it was 176,436 ± 4960, with a significant difference between ORD and DH (*p* = 0.009).

The percentage of admissions transformed into day hospital increased for residents, while for mobility admissions, the percentage increased for ordinary hospitalizations.

The rate of hospitalization over the years had stabilized around the national average value of 123.15 admissions per year per 1000 inhabitants: it was higher in Campania, Aosta Valley, Sardinia, Autonomous Province (A.P.) of Bolzano, and Molise, while it was lowest in Sicily, Veneto, Lombardy, and Piedmont. Regarding acute cases, the number of hospital beds was homogenized around the national average value of 3.0 beds per 1000 inhabitants. They were still higher than the 3.0 standard in Molise, Sardinia, Friuli V.G., Aosta Valley, Umbria, Lombardy, Emilia-Romagna, A.P. Bolzano, and Liguria. It was logical that there was still a weak positive correlation between the hospitalization rate and the number of beds (Spearman’s RHO = 0.4101; *p* = 0.0580): the weakness of the correlation attested a decreasing trend in inappropriate hospitalizations in the various regions. 

Table 2 shows the ORD/DH rate for residents (R) and mobility (M) hospitalization of Italian regions from 2013 to 2017. It should be noted that recourse to day hospital was uneven in the various regions: for residents, it was maximum in Campania (two ordinary admissions for a day hospital) and minimum in Lombardy (five ordinary admissions for a day hospital). The percentage of day hospital was higher for mobility admissions. The Apulian hospitals were the ones that use day hospitals the least. Hospitals in Lombardy, Emilia-Romagna, Umbria, Sicily, and Piedmont also use day hospitals at low rates. The proportions between ORD/DH vary considerably depending on the residents attracted; in particular, in Sardinia, but also A.P. of Trento, Calabria, Piedmont, and Campania, attracted patients were most frequently treated under the ordinary regime, while in Veneto, Friuli-Venezia Giulia, Liguria, Tuscany, Latium, and Abruzzo, the day hospital regime was preferred for them. 

We analyzed the hospitalizations of residents and mobility according to the different types of healthcare facilities: hospitals managed by Local Health Authorities (LHAs), University Hospitals and Polyclinics, IRCCS-Research Institutes, or Private Clinics.

The hospitalization decreased during the studied period by one million. The decrease in admissions of residents from 2013 to 2017 occurred for all types healthcare facilities except for hospitals managed by LHAs. Hospitalizations related to mobility remained stable, but there were increases in hospitals managed by Local Healthcare Authorities and Private Clinics.

Table 3 shows the hospitalizations related to mobility from 2013 to 2107 within the Italian regions, which are divided into Major Diagnostic Categories (MDCs). We had not considered the MDCs related to surgeries that were not related to the diagnosis of discharge (NA) as well as transplants and tracheostomies (PR).

While there was an increase in mobility hospitalizations, in absolute value, from 2013 to 2017, they decreased over our study period, for MDC 1, MDC 2, MDC 3, MDC 5, MDC 14, MDC 15, MDC 16, MDC 17, MDC 21, and MDC 25.

In the studied period, MDC 8 (158,849 ± 4535 admissions) and MDC 5 (64,215 ± 693 admissions) were the first causes of mobility, followed by MDC 1 (46,682 ± 1340 admissions) and MDC 6 (43,497 ± 297 admissions). Lombardy was the region with the highest attraction of MDCs (15 in total), in particular for MDC 5, MDC 13, MDC 1, MDC 9, MDC 6, MDC 10, MDC 17, MDC 11, MDC 4, MDC 23, MDC 14, MDC 12, MDC 21, MDC 18, and MDC 15. Emilia-Romagna had the highest number of attractions for four MDCs: MDC 8, MDC 3, MDC 20, and MDC 22; Latium (MDC 16 and MDC 19), and Tuscany (MDC 2 and MDC 24) had two, while Veneto (MDC 7) and Campania (MDC 25) had one. From 2013, the major attractions were for MDC 8 from Emilia-Romagna (36,318 ± 652) and Lombardy (28,823 ± 2863). 

**Figure 1:**
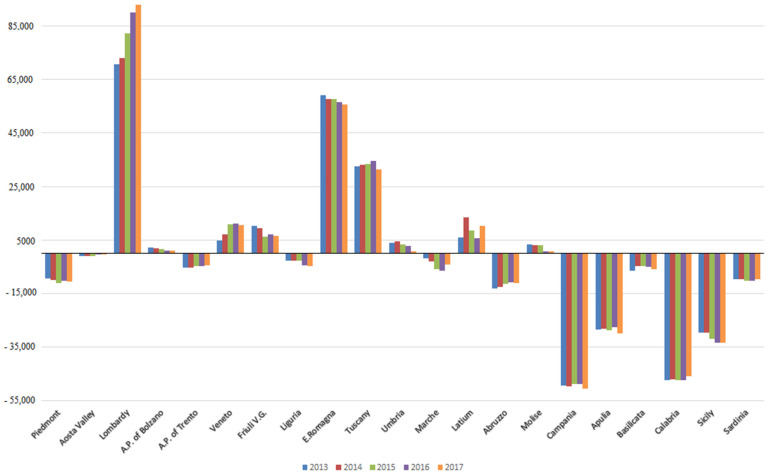
Mobility balances (A − E) of Italian Regions, from 2013 to 2017.

Figure 1 shows the mobility balances (A − E) of Italian regions, from 2103 to 2017.

The southern regions, with the exception of Molise, had negative balances (especially Calabria and Campania). Lombardy was the region with the largest surplus, increasing from 2013 to 2017, followed by Emilia-Romagna and Tuscany. Among the northern regions, Piedmont, Liguria, and A.P. of Trento had negative balances.

We made cartographic representations of the mean of Internal Demand Satisfaction Indexes (IDSI), Attraction Indexes (AI), Escape Indexes (EI), Attraction Absorption Indexes (AAI), and Escape Production Indexes (EPI).

**Figure 2:**
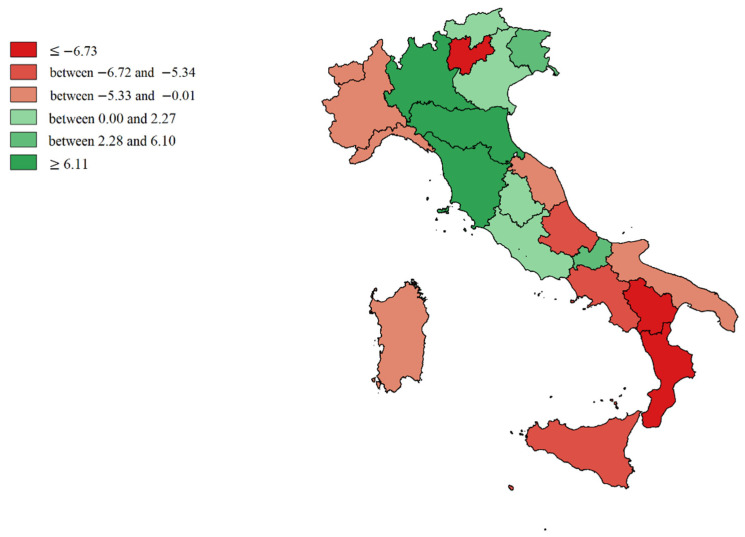
Cartographic representation of Internal Demand Satisfaction Indexes (IDSI), mean from 2013 to 2017.

Figure 2 shows the mean value of the Internal Demand Satisfaction Index (A − E/R + A). The colors shade from intense red (minimum capacity to satisfy internal demand) to intense green (in addition to the capacity to satisfy demand, there was an excess of productivity to be dedicated to attractions). Light green (P.A. of Bolzano, Veneto, Umbria, and Latium) represented the balance point (all the resources potentially dedicated to internal satisfaction, which was also the institutional mission). The southern regions (mainly Calabria and Basilicata), including the Islands and also A.P. of Trento, Abruzzo, Marche, Liguria, Piedmont, and Aosta Valley were more dependent on the hospitals of other regions.

**Figure 3:**
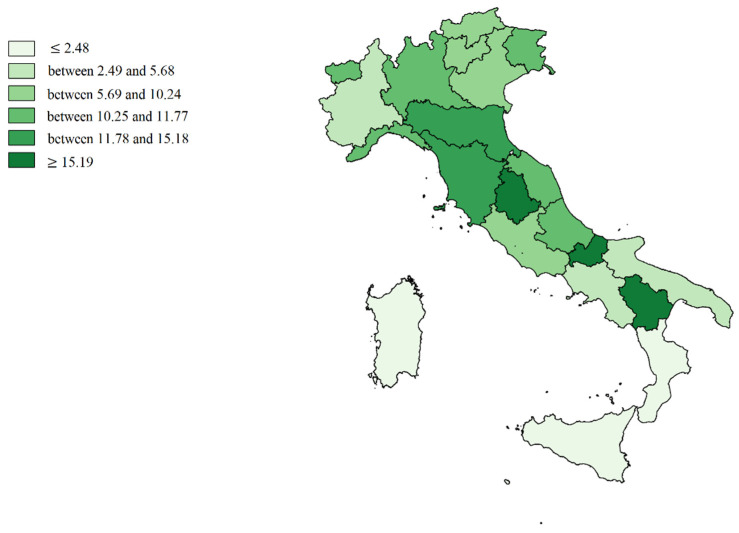
Cartographic representation of regional Attraction Indexes (AI), mean from 2013 to 2017.

Figure 3 shows the mean value of Attraction Indexes (A/R + A): Molise, Basilicata and Umbria are in the first positions (deep green), while the Islands, Calabria, Campania, Apulia, and Piedmont are in the worst positions (soft green).

**Figure 4:**
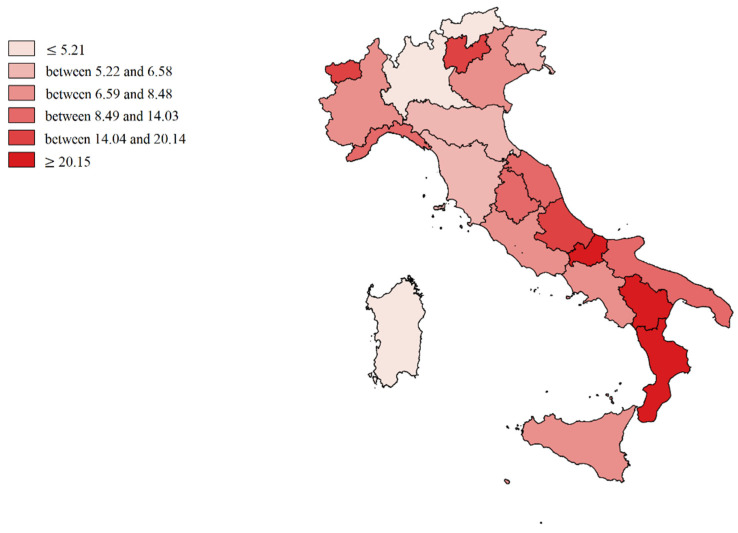
Cartographic representation of Escapes Indexes (EI), mean from 2013 to 2017.

Figure 4 shows the mean value of Escape Indexes (E/R + E): in the most disadvantaged positions are Molise, Basilicata, Calabria, Abruzzo, A.P. of Trento, and Aosta Valley (deep red). Lombardy, A.P. of Bolzano, and Sardinia were the regions that best contain them (light red). 

Small regions with large borders were more sensitive to the phenomenon of mobility, both incoming and outgoing, which is almost physiological for them. It was not a coincidence that several times, the government considered aggregating them into macro regions. A strong negative correlation (Spearman’s RHO = −0.6429; *p* = 0.0017) between IDSI and EI was logical.

**Figure 5:**
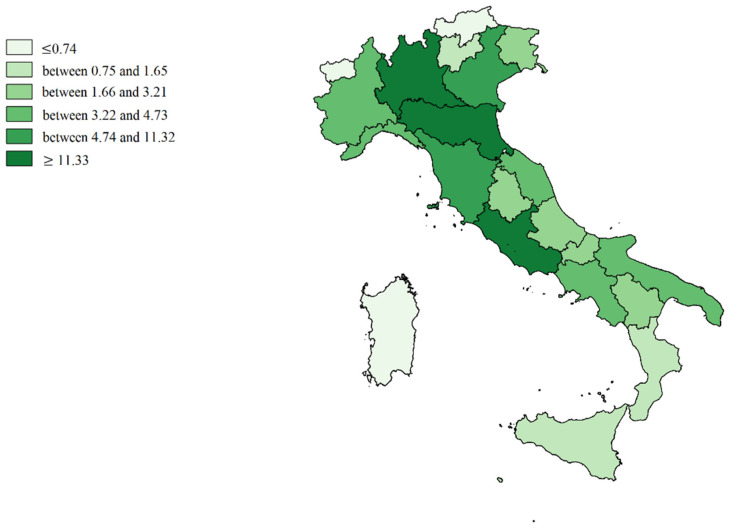
Cartographic representation of regional Attraction Absorption Indexes (AAI), mean from 2013 to 2017.

Figure 5 shows the mean value of Attractions Absorption Indexes (A single Region/Tot. A all Regions), the hospital networks (deep green) of Lombardy, Emilia-Romagna, Latium, Tuscany, and Veneto absorbed more than 60% of all escapes from other regions. Attractions of Aosta Valley, A.P. of Trento, A.P. of Bolzano, Calabria, and the Islands were irrelevant (light green). Therefore, the role played by geographical position on the phenomenon is therefore evident. 

**Figure 6:**
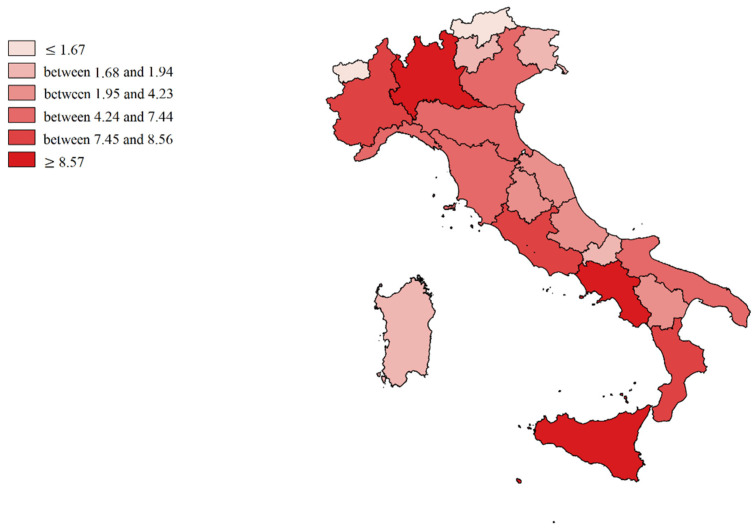
Cartographic representation of regional Escape Production Indexes (EPI), mean from 2013 to 2017.

Figure 6 shows the mean value of Escape Production Indexes (E single Region/Tot. E all Regions). Campania, Latium, Lombardy, Sicily, Piedmont, and Calabria produced the most escapes (deep red), while Aosta Valley A.P. of Bolzano, A.P. of Trento, Friuli-Venezia Giulia, and Molise produced the least (light red). However, these latter regions are the least populous. In fact, we found a positive correlation (Spearman’s RHO = 0,5879; *p* = 0,0051) between AAI and EPI.

**Figure 7:**
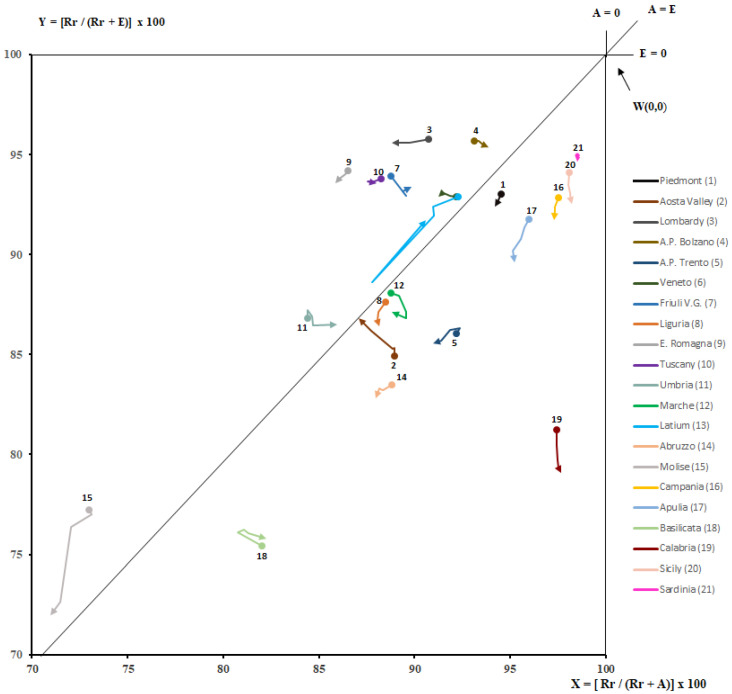
Gandy’s Nomogram of patients’ hospital mobility (upper right quadrant, from 2013 to 2017).

The different capacity of regions to meet the care demand of their own residents and patients from other regions for the period studied was depicted through Gandy’s Nomogram in Figure 7. All the regions were located in the upper right quadrant. This quadrant is divided into two hemi-quadrants, of which the upper one represents the area of optimal planning of public hospital networks: satisfaction of the needs of residents, with a balance sheet (A − E) in surplus. The regions placed in this hemi-quadrant were Lombardy, A.P. of Bolzano, Veneto, Friuli V.G., Emilia-Romagna, Tuscany, Umbria, Latium, and Molise. We could see a clear increase in escapes from 2013 to 2017 for Molise, Campania, Apulia, Calabria, Sicily, and Liguria. In Aosta Valley, we had a decrease in escapes and an increase in attractions. An increase in attractions was mainly found for Lombardy, A.P. of Trento, Veneto, Latium, Emilia-Romagna, and Tuscany. Cuzick’s test was used to evaluate trends of X and Y values for every Italian region. It did not show significant variations over time (*p* > 0.05). This was most likely due to the trends being relatively small and the period under examination being limited.

## 4. Discussion

The topic of inter-regional hospital healthcare mobility is very relevant for several reasons. First, it involves a large number of patients: in 2017 in Italy, the hospital discharges in mobility were 8.52%, which was higher compared to previous years [38]. Some flows can be considered physiological because they are due to movements between neighboring regions or to the size of catchment areas of high specialties. Other flows can be considered “pathological”, because they are due to the qualitative and quantitative insufficiency (real or perceived) of the supply of care in the areas of residence: this has important economic implications, but probably, above all, in terms of the equity of our National Health Service. The aim of this study was to analyze inter-regional healthcare mobility.

All the hospitalizations entered in the HDCs database of the Ministry of Health, which made the data available, were considered.

In the studied period (2013–2017), we found an important decrease in hospitalizations, both in ordinary and day hospital, and a simultaneous increase in inter-regional mobility (+0.90%), while both hospitalization rates and the number of beds in the different regions were homogenized [41].

For residents, the percentage of hospitalizations transformed into day hospitals was increasing. Mobility under the ordinary regime generated the main share of hospitalization, representing on average 74% of hospitalizations compared to 26% for day hospital admissions. The trend for ordinary regime was increasing overall (+1.17%), while day hospital admissions were decreasing. The ORD/DH ratio, for some regions, varied according to whether admissions were residents or attractions.

In a context of general decrease in admissions, hospitalizations in hospitals managed by LHAs and private clinics increased relatively and, we would say, virtuously.

Being able to differentiate the type of hospitalization institution can help identify “quality-driven” mobility (opportunity for patients seeking highly specialized care) and differentiate it from "avoidable" mobility caused by "holes" in the local care offer [45].

According to the Major Diagnostic Categories (MDCs), in Italy, the main causes of hospitalizations in mobility were Diseases and disorders of the musculoskeletal and connective system (MDC 8), Diseases and disorders of the cardiovascular system (MDC 5), Diseases and disorders of the nervous system (MDC 1), and Diseases and disorders of the digestive system (MDC 6). The region with the highest number of MDCs was Lombardy, followed by Emilia-Romagna, Tuscany, and Latium. The biggest attractions were for MDC 8. The general picture of mobility was quite heterogeneous; southern regions had the greatest exports of patients and all showed negative mobility balances except for Molise, which has always had positive balances over the years [46], while the northern and central regions had the greatest attractiveness.

Analyzing the mobility indicators, the regions most able to compensate for their internal demand were A.P. of Bolzano, Veneto, Umbria, and Latium, while patients of the southern regions, Islands, A.P. of Trento, Liguria, Piedmont, Aosta Valley, Abruzzo, and Marche had a “budget” more dependent on hospitals of other regions. According to Attraction Indexes, the southern regions had more disadvantaged positions (with the exception of Molise and Basilicata), while, with regard to Escape Indexes, the northern regions were in a better position (with the exception of A.P. of Trento and Aosta Valley). Only five regions attracted (AAI) more than half of the total healthcare mobility: Lombardy has absorbed the highest percentage, followed by Emilia-Romagna, Latium Tuscany, and Veneto. The territorial dimension of these regions influenced, certainly, the values. It was evident that in small regions, for which almost all territory is “border“, “physiological“ attractions or escapes were more easily produced.

All the regions were, as the logic of NHS warrants, in the upper right quadrant of Gandy’s Nomogram, which expresses the optimal vocation of hospital networks to satisfy first the needs of residents. However, from 2013 to 2017, nine regions (Lombardy, A.P. of Bolzano, Veneto, Friuli V.G., Emilia-Romagna, Tuscany, Umbria, Latium, and Molise) were in the upper “quality” part of the quadrant, where attractions were more than escapes. Southern regions, mainly, have worsened their position in Gandy’s Nomogram.

The Health Pact 2019–2021 aims to counter the phenomenon of extra-regional healthcare mobility, considering it a failure for citizens to move outside the region to achieve better quality and accessibility to care. It is necessary, beyond the physiological mobility, to fill the gaps in supply on the territory. The central government is committed to mapping the flows by type of service, to identifying the correspondence with situations regarding lack of supply, and to drawing up a “Plan to stop” passive mobility, strengthening the supply in the critical sectors. 

Another objective is to counteract providers that act through inappropriate practices outside the regional control.

Bilateral agreements on the supply of significant volumes of services are foreseen as mandatory, according to a model agreed upon by the government and the regions, which must bear, in addition to economic limits, the volumes by type of service and case mix.

The establishment of a healthcare mobility-monitoring observatory is also envisaged to analyze the evolution of this phenomenon and the adequate correspondence between actual and planned volumes of mobility [47].

### Limitations

The study has some limits. (I) The data of HDCs have a long latency time, so we have decided to analyze only consolidated ones that we consider reliable. (II) Border mobility between regions is to be considered “physiological” and does not necessarily identify a choice of trust or mistrust for one region over another but is most often to be attributed to a choice of convenience. In our calculations, no distinction was made between these mobilities, but they were all treated in the same way. However, we hypothesize that the lack of differentiation did not affect the final results, although regions with higher perimeter/area ratios may have been affected more by the lack of this distinction. (III) The small size and low population of some regions limit their potential for care services.

## 5. Conclusions

The regions of central and northern Italy tend to be better able to meet the care needs of their citizens and to attract patients from other regions compared with the southern ones. Despite that, the phenomenon of inter-regional healthcare mobility is continually increasing, yet it is beyond the objectives of this study to offer solutions. It should be a priority to reduce healthcare mobility in its entirety for services of low to medium complexity. This is probably already happening, but since we did not apply our database to the Diagnosis Related Groups (DRG) specification, we cannot document it. We analyzed the phenomenon from an epidemiological point of view and not from an economic–financial one, because we could not assess DRG; thus, the weight of “escapes” and “attractions”.

## Figures and Tables

**Table 1:** Admissions of acute patients to Italian hospitals, 2013–2017.

Year	Residents		Mobility		Total		Total (ORD + DH)
	Ordinary	Day Hospital	Ordinary	Day Hospital	Ordinary	Day Hospital	
2013	6,031,991	2,139,284	498,214	175,638	6.530,205	2,314,922	8,845,127
2014	5,890,301	1,993,205	495,361	183,449	6,385,662	2,176,654	8,562,136
2015	5,780,827	1,868,755	502,274	178,975	6,283,101	2,047,730	8,330,831
2016	5,660,277	1,552,413	507,425	175,832	6,167,072	1,728,245	7,895,947
2017	5,627,008	1,634,522	507,841	168,288	6,134,849	1,802,810	7,937,659

**Table 2:** ORD/DH rate of Italian regions, residents, and mobility, 2013–2017.

Region	2013		2104		2015		2016		2017		Mean	
	R	M	R	M	R	M	R	M	R	M	R	M
Piedmont	3.10	3.83	3.24	4.33	3.44	4.43	3.46	4.68	3.50	4.73	3.35 ± 0.17	4.40 ± 0.32
Aosta Valley	2.21	2.30	2.21	2.21	2.05	2.18	2.42	1.95	2.58	1.71	2.29 ± 0.21	2.07 ± 0.21
Lombardy	5.65	5.18	4.95	4.36	5.01	4.08	4.90	3.97	4.90	4.06	5.08 ± 0.32	4.33 ± 0.44
A.P. Bolzano	3.47	2.82	3.64	2.69	3.73	3.35	3.68	3.65	3.75	3.74	3.65 ± 0.11	3.25 ± 0.43
A.P. Trento	1.96	2.94	1.98	3.28	2.10	3.51	2.23	3.68	2.31	4.08	2.12 ± 0.15	3.50 ± 0.38
Veneto	3.24	2.23	3.35	2.16	3.40	2.32	3.93	2.62	4.81	3.23	3.75 ± 0.65	2.51 ± 0.39
Friuli V.G.	3.34	1.28	3.49	1.37	3.58	1.62	3.74	1.78	3.81	1.68	3.59 ± 0.19	1.55 ± 0.19
Liguria	1.96	1.18	1.99	1.17	2.06	1.17	2.04	1.24	2.81	1.57	2.17 ± 0.36	1.27 ± 0.15
E. Romagna	3.17	3.50	3.28	3.66	3.39	3.66	3.53	3.84	4.67	4.81	3.60 ± 0.61	3.89 ± 0.47
Tuscany	3.14	2.91	3.16	2.85	3.08	2.68	3.10	2.62	3.15	2.61	3.12 ± 0.03	2.73 ± 0.12
Umbria	4.54	3.55	4.85	4.33	4.91	4.90	4.44	4.09	4.22	4.13	4.59 ± 0.29	4.20 ± 0.43
Marche	3.16	3.10	3.25	3.61	3.31	3.95	3.25	3.85	2.97	2.33	3.19 ± 0.13	3.37 ± 0.60
Latium	1.97	1.37	2.06	1.35	2.12	1.37	2.25	1.47	2.26	1.44	2.13 ± 0.12	1.40 ± 0.05
Abruzzo	2.67	2.03	2.73	1.91	3.20	2.25	3.30	2.26	3.45	2.27	3.07 ± 0.35	2.15 ± 0.15
Molise	2.12	2.26	2.07	2.37	2.25	2.27	2.93	2.57	3.04	2.57	2.48 ± 0.46	2.41 ± 0.14
Campania	1.60	1.95	1.70	2.14	1.76	2.23	1.79	2.21	1.84	2.29	1.74 ± 0.09	2.16 ± 0.12
Apulia	3.67	5.08	4.02	5.24	5.19	6.98	6.94	8.76	9.02	9.84	5.77 ± 2.22	7.18 ± 1.88
Basilicata	3.21	2.45	3.29	2.89	3.37	3.24	3.25	3.21	3.53	3.21	3.33 ± 0.13	3.00 ± 0.30
Calabria	2.36	2.36	2.61	2.46	2.77	2.70	3.00	3.25	3.39	4.50	2.83 ± 0.39	3.06 ± 0.79
Sicily	2.52	2.54	3.54	3.68	3.93	3.86	4.03	3.49	4.13	4.16	3.63 ± 0.66	3.55 ± 0.55
Sardinia	2.55	5.30	2.46	5.14	2.41	6.22	2.42	6.05	2.46	5.67	2.46 ± 0.06	5.68 ± 0.42
Total	2.82	2.67	2.96	2.70	3.09	2.79	3.22	2.88	3.44	3.01	3.11 ± 0.24	2.81 ± 0.13

**Table 3:** Hospitalizations in mobility, divided for MDCs, 2013–2017.

*N*	Description	2013	2014	2015	2016	2017
1	Diseases and Disorders of the Nervous System	46,877	47,124	43,643	47,170	46,682
2	Diseases and Disorders of the Eye	30,894	26,780	25,127	25,459	24,362
3	Diseases and Disorders of the Ear, Nose, Mouth and Throat	31,796	32,193	32,167	30,993	30,522
4	Diseases and Disorders of the Respiratory System	26,029	27,582	29,240	27,442	27,590
5	Diseases and Disorders of the Circulatory System	65,500	64,712	63,968	65,735	64,215
6	Diseases and Disorders of the Digestive System	43,024	43,390	43,940	43,590	43,497
7	Diseases and Disorders of the Hepatobiliary System and Pancreas	22,097	22,043	22,318	22,479	22,540
8	Diseases and Disorders of the Musculoskeletal System and Connective Tissue	150,648	157,980	160,552	164,076	160,989
9	Diseases and Disorders of the Skin, Subcutaneous Tissue and Breast	26,211	26,552	27,940	28,133	27,638
10	Diseases and Disorders of the Endocrine, Nutritional and Metabolic System	25,482	25,831	27,284	28,028	29,137
11	Diseases and Disorders of the Kidney and Urinary Tract	32,063	31,856	32,874	32,238	32,744
12	Diseases and Disorders of the Male Reproductive System	15,731	15,725	16,634	16,876	16,273
13	Diseases and Disorders of the Female Reproductive System	31,324	33,592	33,616	34,036	33,498
14	Pregnancy, Childbirth And Puerperium	29,023	29,191	28,527	27,028	25,826
15	Newborn And Other Neonates (Perinatal Period)	4585	4318	4249	3961	3914
16	Diseases and Disorders of the Blood/Blood Forming Organs and Immunological Disorders	7184	6986	6932	6629	6528
17	Myeloproliferative Diseases and Disorders (Poorly Differentiated Neoplasms)	37,233	36,823	36,255	33,857	30,909
18	Infectious and Parasitic Diseases and Disorders (Systemic or unspecified sites)	5659	5875	6208	6235	6379
19	Mental Diseases and Disorders	13,266	13,294	12,484	12,566	13.488
20	Alcohol/Drug Use or Induced Mental Disorders	1261	1211	1234	1333	2137
21	Injuries, Poison, and Toxic Effect of Drugs	5473	5655	5296	5421	5357
22	Burns	366	436	439	410	441
23	Factors Influencing Health Status and Other Contacts with Health Services	14,588	14,697	16,086	16,211	16,457
24	Multiple Significant Trauma	485	523	512	625	623
25	Human Immunodeficiency Virus Infection	1109	986	837	709	708
**Total**		669,921	675,355	678,362	681,240	674,471

## Data Availability

Data obtained from Italian Ministry of Health upon specific request.
